# Recurrent fever leading to the diagnosis of an angiosarcoma of the adrenal gland: a case report

**DOI:** 10.1186/s13256-024-04583-3

**Published:** 2024-05-24

**Authors:** Ann-Kathrin Lederer, Stefanie Zimmer, Rabea Margies, Philipp Krettek, Thomas J. Musholt

**Affiliations:** 1 Section of Endocrine Surgery, Department of General, Visceral and Transplantation Surgery, University Medical Center Mainz, Johannes Gutenberg-University Mainz, Langenbeckstraße 1, 55131 Mainz, Germany; 2 Center for Complementary Medicine, Department of Medicine II, Medical Center, University of Freiburg, Faculty of Medicine, 79106 Freiburg, Germany; 3 Department of Pathology, University Medical Center Mainz, Johannes Gutenberg-University Mainz, 55131 Mainz, Germany; 4 Department of Diagnostic and Interventional Radiology, University Medical Center Mainz, Johannes Gutenberg-University Mainz, 55131 Mainz, Germany

**Keywords:** Sarcoma, Adrenal gland, Neoplasm, Rare disease, Cancer, Soft tissue, Epitheloid

## Abstract

**Background:**

Angiosarcoma of the adrenal gland is a very rare malignant vascular neoplasm. The clinical symptoms are atypical or completely absent. Angiosarcomas of the adrenal gland are therefore often discovered incidentally, and the diagnosis is made histologically after resection.

**Case presentation:**

A 46-year-old white Spanish male who was a previous smoker and nondrinker and was slightly overweight (92 kg, 176 cm, body mass index 29.7 kg/m^2^) with no relevant medical history presented to the internal medicine emergency department of our hospital with an unclear 12 cm tumor of the right adrenal gland. Prior to the computed tomography scan, he had had persistent evening fevers for 4 months and unintentional weight loss of 5 kg. The laboratory results showed anemia and an elevated C-reactive protein, but no hormone production. We performed an open adrenalectomy of the right adrenal gland. Finally, the histologic findings revealed an angiosarcoma of the adrenal gland.

**Conclusion:**

Even though angiosarcomas of the adrenal gland are rare, the differential diagnosis of an angiosarcoma should be considered if a malignant tumor of the adrenal gland is suspected. Treatment decisions should be made on an interdisciplinary basis and preferably in a specialized center. Owing to the rarity of angiosarcomas of the adrenal gland, it is necessary to continue to share clinical experience to gain a better understanding of this particular tumor entity.

## Background

Angiosarcomas are malignant, vascular subtypes of soft tissue neoplasms that account for < 2% of sarcomas [1, 2]. A particularly rare form of angiosarcoma is angiosarcoma of the adrenal gland, which has only been reported in less than 100 patients worldwide [1, 3]. As short reminder, the adrenal gland is a paired endocrine gland located in the retroperitoneum above the kidneys. Each adrenal gland consists of a steroid hormone-producing cortex and a catecholamine-producing medulla, but angiosarcomas of the adrenal gland are usually not hormone-producing tumors, unlike tumors of the adrenal gland itself [4, 5]. The diagnosis of an adrenal angiosarcoma is challenging, as there is a variety of much more common benign and malignant lesions that show similar clinical and imaging behavior [2, 3]. Typical symptoms for the presence of an adrenal angiosarcoma appear to be absent, and the differential diagnosis of angiosarcoma of the adrenal gland is often not considered owing to its rarity. Therefore, this research work aims to give an overview of the angiosarcoma of the adrenal gland with the help of a case report.

## Case presentation

In accordance with the case report (CARE) guidelines [6], this report describes a patient with a histologically proven angiosarcoma of the adrenal gland who underwent an open adrenalectomy at the department of general, visceral, and transplantation surgery of the University Medical Center of Mainz, Germany.

### Chief complaints

A 46-year-old white Spanish male, who was a previous smoker and nondrinker and was slightly overweight [92 kg, 176 cm, body mass index (BMI) 29.7 kg/m^2^] presented to the internal medicine emergency department of our hospital with an unclear 12 cm tumor of the right adrenal gland and an unclear 2.5 cm tumor of the left adrenal gland.

### History of present illness

**Fig. 1:**
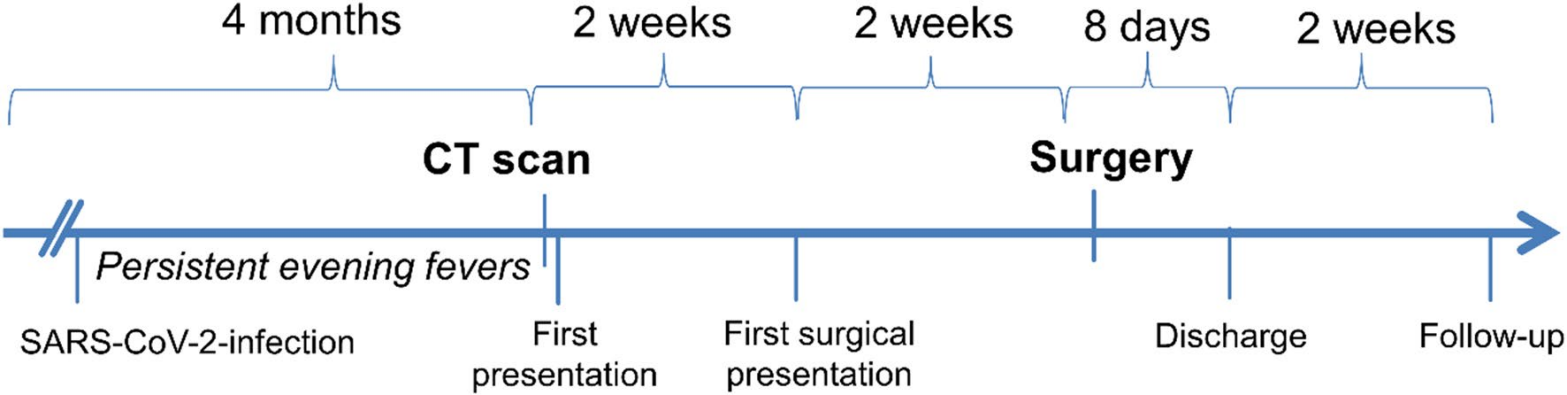
Timeline of the patient

Prior to the computed tomography (CT) scan, which was indicated by the patient’s family doctor, there had been persistent episodes of fever in the evenings for 4 months (Fig. 1). Additionally, an unintended weight loss of 5 kg was reported. Shortly before the onset of the fever episodes, an infection with severe acute respiratory syndrome coronavirus 2 (SARS-CoV-2) had been present. No further complaints were reported. There was no abdominal pain. Food intake was possible without restriction. Bowel movements and urination were regular and possible without discomfort.

### History of past illness and personal and family history

Chronic preexisting conditions and allergies were denied. There was no need for a long-term medication. In the past, a pilonidal sinus had been surgically treated. The family’s medical history was unremarkable. Previous exposure to radiation or chemicals was not recalled.

### Physical examination

Physical examination revealed that the patient was in good clinical condition, with regard to his nutritional status, he was slightly overweight.

He had tachycardia (107 bpm), he was slightly hypertonic (140/70 mmHg), and his respiratory rate was 20 breaths/minute. Further cardiopulmonary examination did not reveal pathological findings.

No cervical lymph node or thyroid gland enlargement was noted, his skin and visible mucous membranes were without lesions, and there was no exanthema.

There was no abdominal pain, no tenderness, and no resistance.

Neurological grossly orienting was also unremarkable.

### Echocardiogram

Sinus rhythm, normal conduction, and sinus tachycardia were noted, and no relevant excitation recovery disorders were detected.

### Pathologic laboratory values

The patient’s hemoglobin was 12.1 g/dl (normal range 13.5–17.5 g/dl).

Mean corpuscular volume (MCV) was 78.5 fl (normal range 83–100 fl).

Mean corpuscular hemoglobin (MCH) was 26.0 pg (normal range 27–33 pg).

International normalized ratio (INR) was 1.3 (normal range = 1.0).

C-reactive protein (CRP) was 140 mg/l (normal range < 5 mg/l).

Endocrinological diagnostics ruled out the presence of a hormone-producing tumor.

### Imaging examinations

**Fig. 2:**
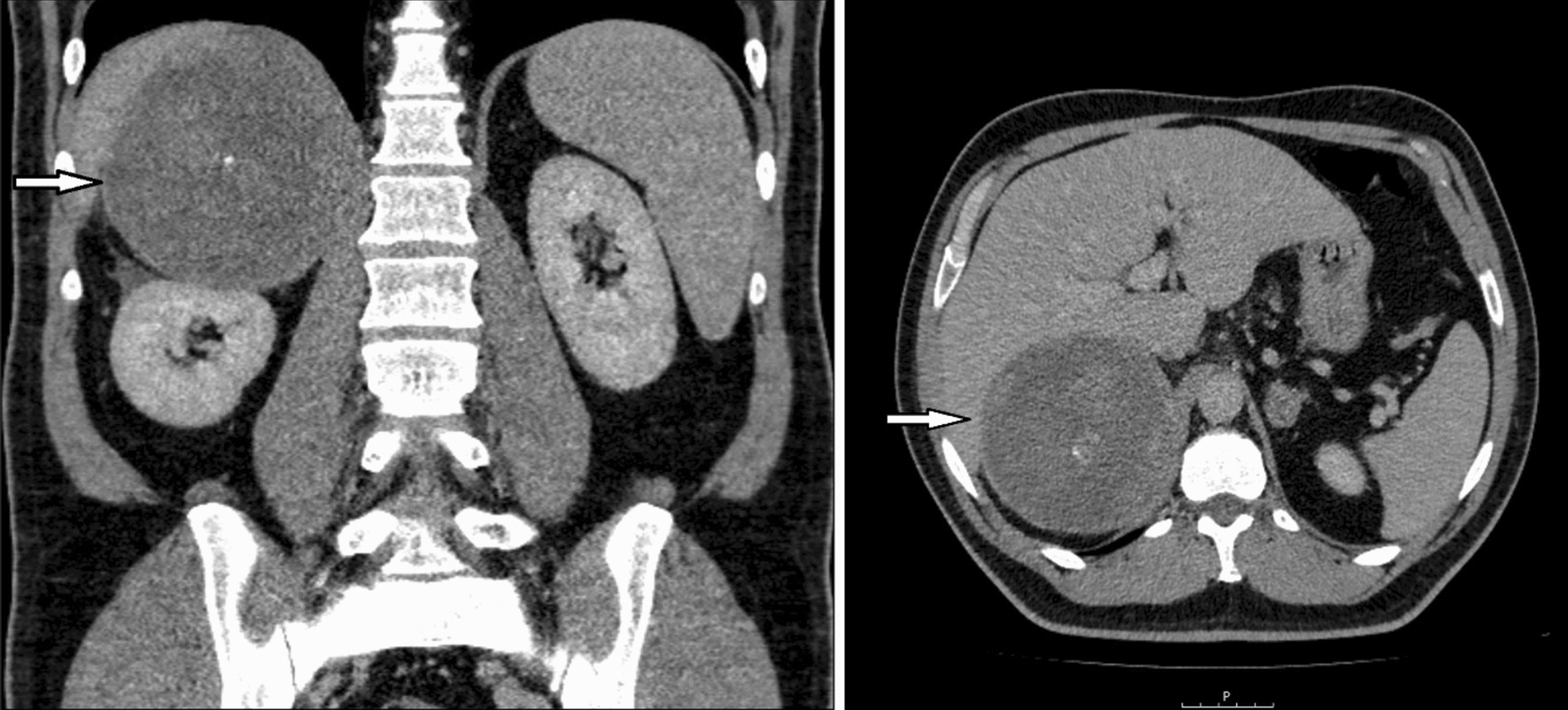
Contrast-enhanced computed tomography scan showing a heterogeneous mass (11.5 × 10.5 cm, marked by arrows) in the right adrenal gland with a central calcification

The contrast-enhanced CT scan demonstrated a heterogeneous mass (11.5 × 10.5 cm) in the right adrenal gland with a central calcification (Fig. 2). In addition, there was a 2.5 cm mass in the left adrenal gland with no signs of malignancy. There was no evidence of distant metastases in either the thorax or the abdomen.

### Treatment

The patient’s case was previously discussed in our interdisciplinary tumor board. As it was not possible to determine with certainty what type of tumor it was and to prevent the spread of tumor cells through biopsy, a complete resection was recommended. Surgical presentation led to the indication for open adrenalectomy owing to tumor size in accordance with the German guideline [7]. The patient consented to surgery.

The operation was performed electively under general anesthesia 1 week after the surgical presentation (Fig. 1). Surgery was performed in supine position via right costal arch incision with an additional extension cranially to the xiphoid. The tumor was found dorsal to the right lobe of the liver stretching and compressing the liver. Inspection of the abdomen revealed no further pathology and no peritoneal metastases. Mobilization of the liver was challenging owing to tumor size and its adherence to the adjacent tissue. We performed a subtle dissection to detach the tumor step by step. To ensure tumor integrity, partial decapsulation of the liver was necessary as a part of the preparation. Luckily, the vena cava and the right kidney were not infiltrated by the tumor. It was possible to remove the tumor completely with an intact capsule, which was later confirmed by pathology. At the end of the procedure, the left adrenal gland was examined. Owing to the nature and size of the left-sided tumor, there was no indication for resection of the left adrenal gland. An adrenal incidentaloma is to be assumed.

The operation lasted 150 minutes. Blood loss can be estimated at around 100 ml. The patient was taken to the post anesthesia care unit after operation. After a short monitoring phase, the patient was transferred to the normal ward on the day of surgery. On the second postoperative day, the patient complained shortness of breath, which is why a CT scan was performed to rule out a pulmonary embolism. The CT scan revealed suspected postoperative pneumonia. Antibiotic therapy was started. The patient received intensified respiratory training. Afterwards, the patient did well and was discharged on the eighth postoperative day.

### Pathological examination and final diagnosis

**Fig. 3:**
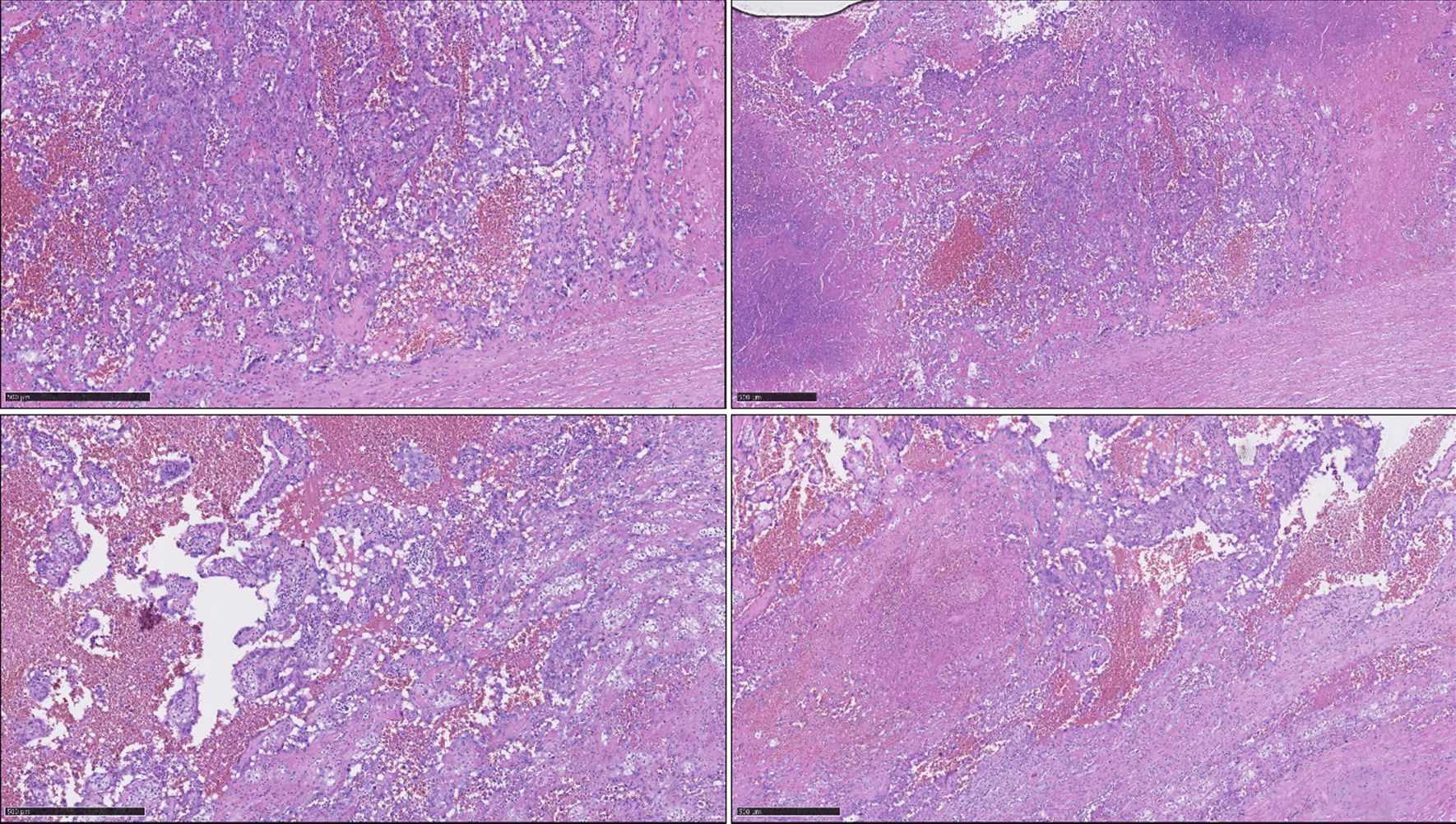
Histological findings (hematoxylin and eosin stain)

Histologically, the nodule of the right adrenal gland measuring 14 × 12 × 11 cm consisted of bulging elastic tissue. The nodule showed a centrally strongly softened, reddish-blackish, macroscopically predominantly necrotic surface with somewhat firmer areas at the margins. The nodular lesion was completely surrounded by a fibrous capsule, and the yellowish adrenal parenchyma was sparse and marginalized. Microscopically, only few areas with vital epitheloid cells with variations in nuclear size, occasional mitoses, and increased apotosis were found. The cells formed partly solid, partly pseudoglandular, and anastomosing structures, some of which were filled with erythrocytes and granulocytes (Fig. 3).

**Fig. 4:**
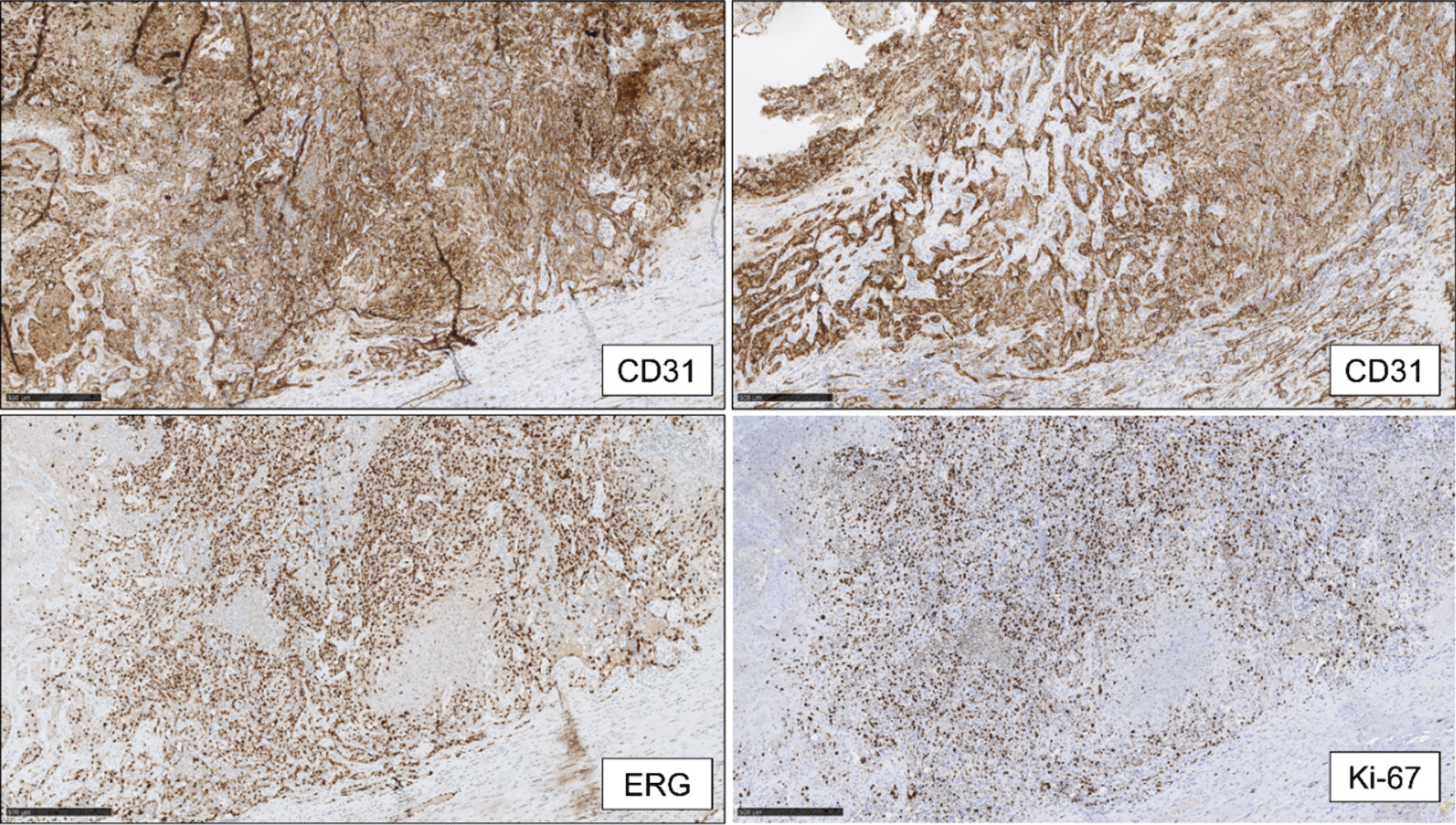
Findings of immunohistochemistry

Immunohistochemically, the epitheloid cells showed a strongly increased proliferation (Ki-67 90%) with a strong expression of erythroblast-transformation-specific-related gene (ERG), platelet endothelial cell adhesion molecule (CD31), cytokeratin AE1/AE3, and, only sporadically, CD34, leading to the diagnosis of a highly proliferative vascular tumor (Fig. 4).

We found no mismatch repair gene deficiency (pMMR/MSS) and no Her2 amplification. PD-L1 expression of the tumor proportion score, the combined positive score, and the immune cell score were 30%, 40%, and 10%, respectively. Molecular pathology of the tumor tissue in the RNA-based next generation sequencing analysis (Archer Fusion*Plex* Sarcoma NGS Panel) revealed no fusion in the examined gene regions. Finally, the findings were compatible with a completely resected angiosarcoma of the right adrenal gland.

### Outcome and follow-up

The patient was seen again 2 weeks after surgery. Physical examination showed normal findings. The recovery went well. In accordance with the recommendation of the postoperative interdisciplinary tumor board, the patient was advised to attend the sarcoma outpatient clinic at our hospital for evaluation of adjuvant radiation and chemotherapy.

## Discussion and conclusion

This case report is intended not only to illustrate the case of a rare tumor entity, the angiosarcoma of the adrenal gland, but also to discuss the reported diagnostic and therapeutic strategy critically. Adrenal angiosarcoma appears to occur more frequently in men and is more likely to be a disease of the elderly [1, 3]. Our patient was male, but not even 50 years old. There were no anamnestic risk factors for the development of an angiosarcoma. Most angiosarcomas arise spontaneously, but the occurrence is reported to be associated with several hereditary risk factors (e.g., neurofibromatosis) and tissue-damaging factors, such as radiation or exogenous toxins [2]. A small number of scientific publications have dealt with the role of the breast cancer gene (*BRCA*) in angiosarcomas, as case reports and case series indicate the occurrence of cutaneous angiosarcomas in *BRCA* carriers after treatment for breast cancer [8–11]. It is known that mutations in the tumor suppressor genes *BRCA1* or *BRCA2* contribute to the development of breast and ovarian cancer [11–13]. Kadouri *et al*. reported an angiosarcoma rate of 0.43% in patients with a genetic predisposition (*BRCA1*, *BRCA2*, p53 mutation) who underwent radiation owing to breast cancer [9]. However, Schlosser *et al*. reported no cases of sarcoma after evaluating 266 breast cancer patients who were *BRCA* carriers and had undergone radiation [14]. Although the role of *BRCA* in the development of sarcomas is plausible, especially after radiation, sarcomas appear to be very rare in the cohort of *BRCA* carriers. In general, the role of genetic and external predisposition in the development of angiosarcoma of the adrenal gland remains unclear, as most case reports deny the exposure to risk factors of affected patients [15]. Angiosarcomas originate in lymphatic or blood vessels, which is why they can develop in any soft tissue. Thus, recent publications discuss whether the tissue of origin affects the biology of the tumor and the prognosis of the patient [2]. Owing to the rarity, it is difficult to make a valid statement about an individual prognosis of an adrenal angiosarcoma. Ladenheim *et al*. evaluated survival data of 40 adrenal angiosarcoma patients and calculated a 5-year survival rate of 30% [1]. Angiosarcomas in general are reported to have an aggressive tumor biology and to be prone to rapid recurrence and distant metastases [2]. The 5-year survival rate of all types of angiosarcomas is reported to be only 30% [16]. Angiosarcomas are considered to be chemotherapy-sensitive, which opens up the possibility of medicinal treatment [17]. In our case, the interdisciplinary tumor board recommended adjuvant chemotherapy and radiation, as the tumor posed a high risk of recurrence owing to its size. Conforti *et al*. reported that patients with a tumor larger than 5 cm might benefit from chemotherapy [18]. However, the overall evidence is lacking regarding chemotherapy in angiosarcoma, especially in adrenal angiosarcoma [19, 20].

The clinical appearance of angiosarcoma of the adrenal gland is often inconspicuous, which can delay the diagnosis. Other case reports emphasize that adrenal angiosarcomas are mostly incidental findings [1]. Angiosarcomas of the adrenal gland do not produce hormones, which is why no specific symptoms arise that could indicate an adrenal tumor [4, 5]. Owing to the tumor size of an often already advanced angiosarcoma, which may lead to an abdominal displacement, some patients experience nonspecific abdominal pain and discomfort [20–22]. Fever, as occurred in our patient, is not a classic sign of adrenal angiosarcoma. We found only one other case of angiosarcoma of the adrenal gland with a slight fever episode [23]. However, fever may be a possible symptom of angiosarcoma in general, as other publications have described fever as a symptom of angiosarcoma at other sites [24, 25]. It is known that fever can occur with neoplasia, but it is not a common symptom of malignant diseases [26]. Our patient still had subfebrile temperatures shortly after the operation. Later on, the fever episodes disappeared, which indicates a possible association between the tumor and the fever episodes. To be honest, even though we expected a malignant tumor owing to the imaging and the tumor size, we did not think of an adrenal angiosarcoma. We also considered a hemorrhage of an adrenal tumor as a differential diagnosis, consistent with the patient’s preoperative anemia. CT scan revealed a heterogeneous tumor mass with central calcifications, both suspicion signs for malignancy, but not specific for angiosarcomas [5, 27]. An angiosarcoma does not necessarily show signs of malignancy on imaging, as reported by Yang *et al*., for example, who misinterpreted an angiosarcoma as an adenoma [28]. According to the guidelines, there is a risk of malignancy of around 25% if the tumor is larger than 6 cm [5, 7, 29]. Thus, in the case of surgery, it is all the more important to aim for a complete removal of large adrenal tumors without damage to the tumor capsule. In accordance with the guidelines for adrenal tumors, we opted for an open adrenalectomy with resection of the retroperitoneal adipose tissue and readiness for multivisceral enbloc resection if necessary [7]. There is much debate about the extent of surgical resection in the field of soft tissue sarcomas. Earlier research suggests that wider resection margins are associated with a prolonged disease-free survival, whereas more recent research indicates that only the quality of the resection and not the extent might be essential for disease recurrence [30, 31]. In our patient, the critical resection margins were the vena cava and the diaphragm. However, if we had wanted to achieve a wider resection margin here, a complex reconstruction of the diaphragm and the vena cava would have been necessary, which would have been associated with increased morbidity of the patient. In general, our patient was in a difficult situation owing to the occurrence of bilateral tumors of unknown dignity. Benign adrenal tumors occur bilaterally in about 15% of patients, while bilateral adrenal metastases are common in metastatic cancer and occur in more than 40% of patients [32]. To date, there have been no reports of bilateral angiosarcomas, and the bilateral occurrence of angiosarcomas, although not impossible, seems very unlikely owing to the rarity of the disease and the lack of known genetic factors for the development of angiosarcomas of the adrenal gland. However, in 2012, Lepoutre-Lussey *et al*. reported the case of a patient with an adrenal angiosarcoma who also suffered from a hormone-producing adrenocortical adenoma [33]. The guideline of the European Society of Endocrinology recommends the individual evaluation of each tumor in the case of bilateral adrenal tumors [34]. As already mentioned, surgery on the right adrenal gland was indicated on the basis of the diagnostic results of our patient. On the left side, there was no suspicion of malignancy either on imaging or intraoperatively, which is why we decided against resection. The indication for adrenalectomy should be critically evaluated, as there is a risk of adrenal insufficiency and a subsequent hypocortisolism, which leads to lifelong substitution of glucocorticoids and mineralocorticoids and bears the risk of a life-threatening adrenal crisis [35]. Bilateral adrenal surgery is associated with a high risk of adrenal insufficiency, even in the case of a partial adrenalectomy [36].

Unlike for most types of soft tissue sarcomas, biopsy is not a standard procedure for primary adrenal tumors [7, 17, 37]. The histologic analysis of adrenal tumors is challenging, which is emphasized by Duregon *et al*., who evaluated diagnostic certainty of 300 cases with an adrenocortical carcinoma. Almost 10% of cases were misdiagnosed, including also three cases of an adrenal angiosarcoma [38]. As in our case, most angiosarcoma have epitheloid morphology [1]. Typically, an expression of ERG, CD31, and cytokeratin AE1/AE3 can be found, which has been reported in several adrenal angiosarcoma case reports [1, 20, 28, 39–41]. Angiosarcomas show a strongly increased proliferation measured by the Ki-67 index, but this is not specific. Diagnostically, the staining of steroidogenic factor 1 (SF-1) could have been considered, but SF-1 is currently not part of our routine diagnostics. SF-1 is able to differentiate malignant adrenocortical tumors [42]. Overall, it has to be said that the preoperative knowledge of the diagnosis of an angiosarcoma would probably not have changed our surgical strategy.

The case emphasizes the importance of interdisciplinarity. In addition to visceral surgery, radiology, pathology, and oncology have also contributed to the diagnosis and the jointly developed treatment strategy for the patient. In retrospect, we should have paid more attention to the patient’s preoperative laboratory values. In terms of a responsible patient blood management, preoperative anemia diagnostics could have helped to better prepare the patient for the operation [43]. The preoperative laboratory values indicated iron-deficiency anemia, which can be easily treated prior to surgery [44]. Unexplained anemia might be a warning sign, albeit unspecific, that has been reported in some adrenal angiosarcoma patients [3, 20, 45]. Furthermore, in contrast to our patient, many of the adrenal angiosarcoma patients reported in case reports suffered from flank or abdominal pain [3, 20, 27, 28, 39, 40, 45, 46].

As a conclusion, the angiosarcoma of the adrenal gland is a rare tumor entity with unspecific symptoms. Angiosarcomas should be considered as a differential diagnosis if a malignant tumor of the adrenal gland is suspected, especially if there is no production of hormones. Treatment at sarcoma centers is desirable, as recommended in the guidelines for treatment of soft tissue sarcomas. Owing to the rarity of angiosarcomas of the adrenal gland, it is necessary to continue to share clinical experience to gain a better understanding of this particular tumor entity.

## Data Availability

Data are available on reasonable request by the corresponding author.
